# Effect of a fall within three months of admission on delirium in critically Ill elderly patients: a population-based cohort study

**DOI:** 10.1007/s40520-024-02740-8

**Published:** 2024-05-14

**Authors:** Fan Bu, Hong-tao Cheng, Zi-lin Wang, Yu-shan Hou, Zhuang Zhuang, Can-yang Li, Ya-qi Wang, Yue Zhang, Jun Lyu, Qi-yuan Lyu

**Affiliations:** 1 School of Nursing, Jinan University, Room 1015, Guangzhou, China; 2 Department of Geriatric Psychology, Shandong Daizhuang Hospital, Jining, China; 3 Department of Clinical Research, The First Affiliated Hospital of Jinan University, Guangzhou, China; 4 Guangdong Provincial Key Laboratory of Traditional Chinese Medicine Informatization, Guangzhou, China

**Keywords:** Older adults, History of falls, Delirium, Mortality, Intensive care unit, Pressure injury

## Abstract

**Background:**

Delirium is common among elderly patients in the intensive care unit (ICU) and is associated with prolonged hospitalization, increased healthcare costs, and increased risk of death. Understanding the potential risk factors and early prevention of delirium is critical to facilitate timely intervention that may reverse or mitigate the harmful consequences of delirium.

**Aim:**

To clarify the effects of pre-admission falls on ICU outcomes, primarily delirium, and secondarily pressure injuries and urinary tract infections.

**Methods:**

The study relied on data sourced from the Medical Information Mart for Intensive Care IV (MIMIC-IV) database. Statistical tests (Wilcoxon rank-sum or chi-squared) compared cohort characteristics. Logistic regression was employed to investigate the association between a history of falls and delirium, as well as secondary outcomes, while Kaplan–Meier survival curves were used to assess short-term survival in delirium and non-delirium patients.

**Results:**

Study encompassed 22,547 participants. Delirium incidence was 40%, significantly higher in patients with a history of falls (54.4% vs. 34.5%, p < 0.001). Logistic regression, controlling for confounders, not only confirmed that a history of falls elevates the odds of delirium (OR: 2.11; 95% CI: 1.97–2.26; p < 0.001) but also showed it increases the incidence of urinary tract infections (OR:1.50; 95% CI:1.40–1.62; p < 0.001) and pressure injuries (OR:1.36; 95% CI:1.26–1.47; p < 0.001). Elderly delirium patients exhibited lower 30-, 180-, and 360-day survival rates than non-delirium counterparts (all p < 0.001).

**Conclusions:**

The study reveals that history of falls significantly heighten the risk of delirium and other adverse outcomes in elderly ICU patients, leading to decreased short-term survival rates. This emphasizes the critical need for early interventions and could inform future strategies to manage and prevent these conditions in ICU settings.

**Supplementary Information:**

The online version contains supplementary material available at 10.1007/s40520-024-02740-8.

## Background

Falls have become the second leading cause of death worldwide [1]. In the United States, approximately 30% of people over the age of 65 experience a fall each year [2]. Falls not only cause a variety of serious consequences, such as disability, loss of autonomy, and serious neurological damage, but they also increase the risk of death [3, 4]. Across all ethnic groups, falls are responsible for a staggering 75% of unintentional deaths in people over the age of 70 [5]. The financial burden of falls in the U.S. reached approximately $5 billion in 2015, imposing significant financial costs on the healthcare infrastructure [6]. Therefore, early detection and prevention of falls are essential to promote healthy aging.

Delirium, characterized by acute neuropsychiatric symptoms such as inattention and sudden cognitive decline, is notably prevalent among elderly inpatients, with incidences reaching 70–87% [7]. This condition, often overlooked in intensive care unit (ICU) settings due to preexisting conditions in the elderly, can lead to severe consequences including functional impairment, extended hospital stays, cognitive decline, and heightened mortality rates—2–4 times higher than non-ICU settings [8–10]. At the same time, the management of delirium is associated with high healthcare costs and caregiver burden, the cumulative cost over 30 days for ICU patients with delirium was $17,838 [11]. This figure is expected to increase as the population continues to age. However, early detection and appropriate care can prevent or mitigate delirium in geriatric patients [12], potentially reducing healthcare costs, length of hospital stays, and mortality.

While previous literature has recognized the association between falls and delirium [13], previous studies often included a history of falls as part of a cluster of risk factors without isolating it as a standalone variable. prior investigations have not specifically focused on the unique setting of the ICU.

While prior studies have explored the link between fall history and delirium, they typically treat fall history as part of a broader set of risk factors without specific focus on the ICU context or precise timing of falls. This overlooks the unique dynamics of falls within the ICU setting and their temporal proximity to ICU admission. Moreover, existing research primarily delves into fall risk factors and general outcomes post-fall, rarely connecting falls directly to pressure injury or UTI, especially within the ICU context. ICU patients are more prone to severe outcomes like delirium due to their critical health and complex treatments such as mechanical ventilation [14]. This study advances the understanding of falls' impact on ICU patients by isolating fall history as a unique variable and exploring its link to specific outcomes like delirium, pressure injuries and urinary tract infections (UTIs). Furthermore, this study integrates survival analysis to explore how pre-admission falls impact mortality and severe outcomes, like delirium, in elderly ICU patients. By focusing on this unique environment, the research aims to provide vital insights into early intervention strategies, significantly enriching the current body of knowledge regarding patient care in critical care settings.

## Methods

### Study design and population

The study relied on data sourced from the Medical Information Mart for Intensive Care IV (MIMIC-IV) database, version 2.0, which is an extensive, freely accessible, and publicly available resource [15]. The MIMIC-IV database received funding support from the National Institute of Biomedical Imaging and Bioengineering (NIBIB) of the National Institutes of Health (NIH) under grant numbers R01-EB001659 (2003–2013) and R01-EB017205 (2014–2018). Approval for the use of the database was obtained from the institutional review boards of Beth Israel Deaconess Medical Center (Boston, MA) and the Massachusetts Institute of Technology (Cambridge, MA). This study was an analysis of the third-party anonymized publicly available database with pre-existing institutional review board (IRB) approval. The MIMIC-IV database comprehensively captures general patient information, disease-related data, relevant treatments administered, and patients' key blood biochemical indices [16, 17].

**Fig. 1:**
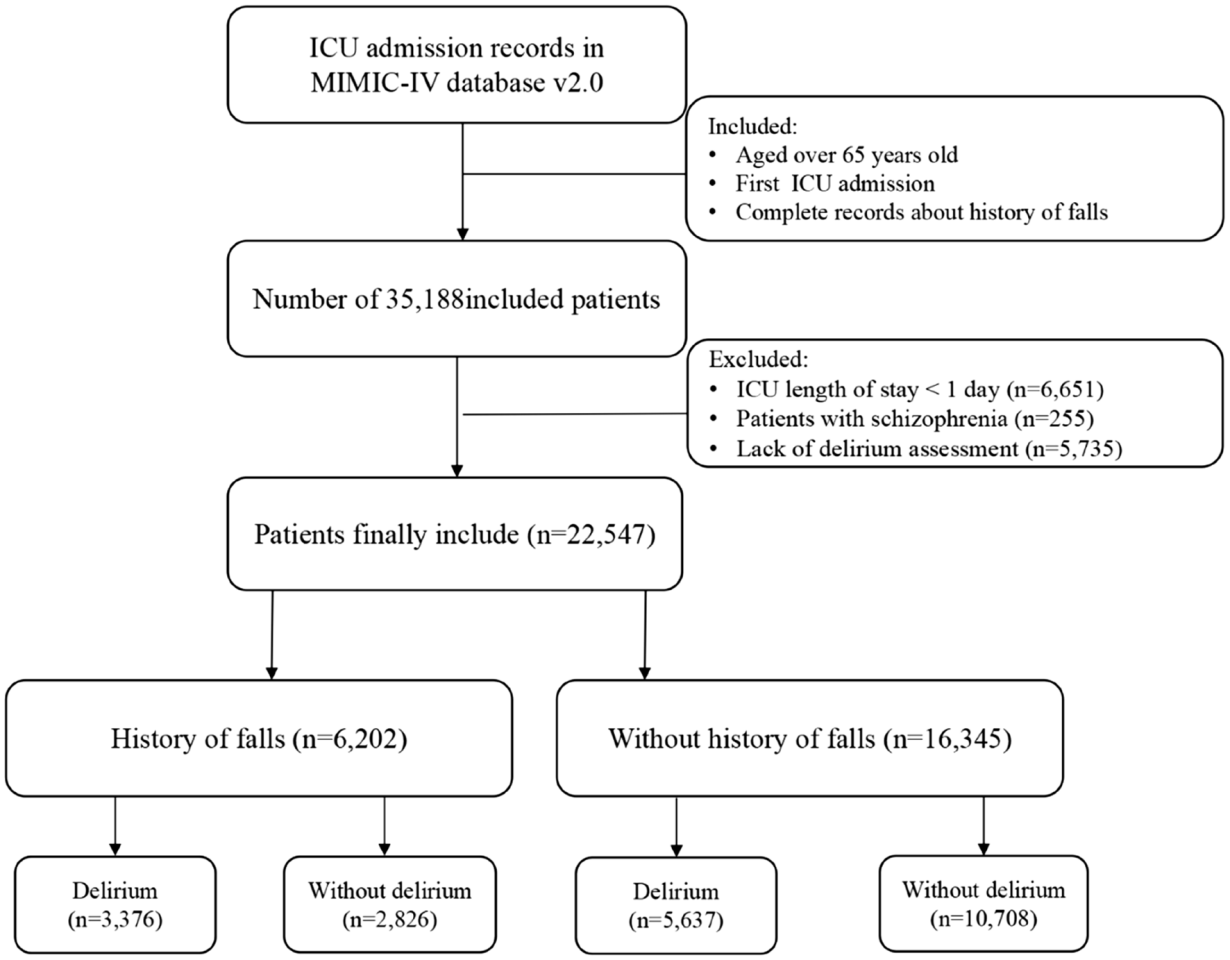
Flowchart of Delirium in Study Participants

The subject selection criteria for this study were clearly defined: patients age 65 or older in whom a history of falls in the last 3 months was addressed were included. However, individuals with an ICU stay of less than one day or those without documentation of delirium and severe mental illness were intentionally excluded from this study. Ultimately, a cohort of 22,547 patients was captured in this investigation (Fig. 1).

### Exposure and outcomes

Falls represent unanticipated occurrences beyond an individual's control, characterized by abrupt, involuntary, and unintended shifts in a patient's position resulting in descent to the ground or a lower level compared to their initial position [4]. The exposure factors in this study were defined as the incidence of unintentional falls among elderly, critically ill patients in the three months prior to ICU admission. A patient's fall history was meticulously assessed upon admission by trained ICU staff through a direct question: "Have you had a fall in the past three months?" An affirmative response to this question identified the patient as having a recent fall history.

Delirium, also referred to as acute encephalopathy, is characterized by changes in consciousness, attention impairment, and cognitive dysfunction [18]. We, employing the Confusion Assessment Method in the ICU (CAM-ICU) [19] and a review of physician and nursing notes [20], conducted the delirium assessment. The assessment of patients' mental status changes during their hospital stay was conducted through a comprehensive review of medical records, including initial and daily observations recorded by nursing staff, evaluations documented in progress notes by nurses or physicians specialized in delirium management, insights from specialized consultations, and summaries provided at discharge. This crucial information is meticulously documented within the "chartevents" form penned by the healthcare provider. Delirium could be defined as a patient who either had 1) a positive CAM-ICU or 2) diagnosed with delirium by a physician based on chart documentation.

Study outcomes included delirium, urinary tract infections and pressure injury, with delirium as the primary outcome and urinary tract infections and pressure injury as secondary outcomes. UTIs were diagnosed according to the International Classification of Diseases, 9th and 10th versions. Pressure injuries were carefully assessed and documented by dedicated intensive care nurses.

### Variable collection

Navicat Premium software, version 16, using Structured Query Language, was used for data extraction. The maximum percentage of missing values for any of the extracted variables did not exceed 20%, and multiple imputation was used to deal with these missing data [21]. The selection of covariates in our study was based on a comprehensive approach that took into account prior literature, clinical expertise, and the availability of data within the database. The following variables were included in this study:

1. General characteristics: age, sex, marital status, race, language, weight, hospital and ICU length of stay.
2. Scores: Glasgow Coma Scale (GCS), Sequential Organ Failure Assessment (SOFA), Braden scores and Charlson Comorbidity Index (CCI).
3. Comorbidities: myocardial infarct, congestive heart failure, dementia, rheumatic disease, mild liver disease, diabetes, renal disease, malignant cancer, severe liver disease, cerebrovascular disease, hypertension, sepsis, depression, pain, paraplegia, peripheral vascular disease and metastatic solid tumor.
4. Vital signs: heart rate mean, mean blood pressure, respiratory rate mean and temperature mean.
5. Laboratory parameters: SpO_2_ mean, albumin, Blood Urea Nitrogen (BUN) and creatinine.
6. Treatments and drugs: enteral nutrition, mechanical thrombectomy, dialysis present, vasoactive agents, sedatives and invasive mechanical ventilation.
7. Outcomes: delirium, pressure injury, urinary tract infection and 30-day, 180-day, and 360-day mortality.

### Covariates

In this study, independent variables with potential impact on outcomes were considered as covariates and adjusted for within regression models based on clinical expertise and literature review. For delirium, adjustments were made for age, sex, race, marital status, dementia, depression, cerebrovascular disease, sedative use, CCI, SOFA, GCS, and Braden scores. For aspiration pneumonia and pressure injuries, adjustments were made for age, sex, race, GCS, CCI, SOFA, and Braden scores.

### Statistical methods

This observational study was conducted in adherence to the STROBE (Strengthening the Reporting of Observational Studies in Epidemiology) guidelines to ensure comprehensive and transparent reporting of the research findings [22]. The study initially performed a descriptive statistical analysis on the collected and organized data. Patients were stratified based on falls presence or absence in the three months before admission. Continuous variables were expressed as medians with interquartile ranges, and differences were compared using the Wilcoxon rank-sum test. Categorical variables were presented as counts and percentages, and group differences were assessed using the chi-squared test. Logistic regression models determined the association between fall history and outcomes, yielding odds ratios (OR) and 95% confidence intervals (CI).

To show the survival rates at 30, 180, and 360 days among the elderly ICU patients who developed delirium, we estimated Kaplan–Meier survival curves and assessed differences in survival distributions between groups using the log-rank test. To examine joint associations, we divided the entire cohort into four groups based on the variables: a history of falls and the presence of delirium. Baseline data for these four groups were tabulated, and between-group differences were assessed using the Kruskal–Wallis H test for continuous variables and the chi-squared test for categorical variables. Hazard ratios (HRs) and 95% confidence intervals for mortality at 30, 180, and 360 days were calculated for the different groups compared with older patients, no history of falls, and no delirium.

Subsequently, this study examined the association between a history of falls and delirium in different subgroups of elderly ICU patients. We examined the association within the subgroups of sex, age, race, marital status, dementia, cerebrovascular disease, and depression. To assess the combined association, we divided the total population into four categories based on history of falls and presence of delirium. The short-term mortality risk in the different groups was calculated in contrast to ischemic stroke patients without fall history and delirium.

Finally, two sensitivity analyses were conducted to assess our findings' robustness. Covariate adjustments were made using propensity score matching (PSM). Propensity scores, derived from logistic regression models incorporating sex, age, race, marital status, body weight, ventilation status, GCS, CCI, and SOFA scores, were estimated. Employing a 1:1 matching algorithm with the nearest neighbor method, the matched population underwent comparison using the Wilcoxon rank-sum test or chi-squared test. To address potential unmeasured confounding factors, the E-value was calculated—an indicator in observational studies gauging the impact of such factors on study outcomes. Higher E-values signify greater result robustness and decreased susceptibility to confounding influences.

Data were processed using R software (version 4.3.0, https://www.r-project.org/). A p-value of less than 0.05 was considered indicative of statistical significance.

## Result

### Baseline characteristics of the study cohort

After careful screening, a total of 22,547 elderly patients were included in the study. Of these, 6,206 patients had experienced a fall within the three months prior to ICU admission. Table 1A,1B,1C,1D describes the baseline characteristics of the study cohort, categorized by exposure factor (Table 1B,1C,1D in Supplementary Materials). Of the total patient population, 12,078 (53.6%) were male, with a median age of 76.71 years (interquartile range: 70.65–83.82 years) and a median ICU length of stay of 2.44 days (interquartile range: 1.60–4.30 days). Compared to patients without a fall history, those with a fall history had an older median age (79.57 vs. 75.78 years), lower weight (73.00 kg vs. 77.80 kg), and a higher proportion of unmarried individuals (including single, divorced, widowed, etc.). These differences were statistically significant (P < 0.05).

**Table 1:** A Univariate Analysis of Demographic Characteristics and Outcomes with and without History of Fall

Variable	Overall (n = 22,547)	Without history of falls (n = 16,345)	History of falls (n = 6202)	P-value
General characteristics				
Age (years old)	76.71(70.65, 83.82)	75.78(70.23, 82.56)	79.57(72.18, 86.67)	< 0.001
Gender (%)				< 0.001
Male/Female	12,078/10469 (53.6/46.4)	8998/7347 (55.1/44.9)	3080/3122 (49.7/50.3)	
Marital status				< 0.001
Married/ Unmarried/unknown	10,644/11903 (47.2/52.8)	7311/9034 (44.7/55.3)	3333/2869 (53.7/46.3)	
Race (%)				0.147
White/Others^&^	15,979/6568 (70.9/29.1)	11,539/4806 (70.6/29.4)	4440/1762 (71.6/28.4)	
Language (%)				0.405
English/ Unknown	20,053/2494 (88.9/11.1)	14,519/1826 (88.8/11.2)	5534/668 (89.2/10.8)	
Weight (kg)	76.45 (64.20, 90.05)	77.80 (65.60, 91.35)	73.00 (61.00, 86.20)	< 0.001
Hospital Los (days)	7.63 (4.86, 12.59)	7.55 (4.87, 12.31)	7.80 (4.83, 12.90)	0.039
ICU Los (days)	2.44 (1.60, 4.30)	2.36 (1.56, 4.19)	2.67 (1.70, 4.69)	< 0.001
Outcomes				
Delirium (%)				< 0.001
Yes/ No	9013/13534 (40.0/60.0)	5637/10708 (34.5/65.5)	3376/2826 (54.4/45.6)	
Pressure injury (%)				< 0.001
Yes/ No	4176/18371 (18.5/81.5)	2727/13618 (16.7/83.3)	1449/4753 (23.4/76.6)	
Urinary tract infections (%)				< 0.001
Yes/ No	4093/18454 (18.2/81.8)	2569/13776 (15.7/84.3)	1524/4678 (24.6/75.4)	
30-day mortality (%)				< 0.001
Alive/ Expired	18,805/3742 (83.4/16.6)	14,007/2338 (85.7/14.3)	4798/1404 (77.4/22.6)	
180-day mortality (%)				< 0.001
Alive/ Expired	16,117/6430 (71.5/28.5)	12,213/4132 (74.7/25.3)	3904/2298 (62.9/37.1)	
360-day mortality (%)				< 0.001
Alive/ Expired	14,838/7709 (65.8/34.2)	11,344/5001 (69.4/30.6)	3494/2708 (56.3/43.7)	

Median and interquartile range (25th and 75th percentiles) were computed for continuous variables, and frequencies and percentages were computed for categorical variables

The Wilcoxon rank-sum test was used to compare group differences for continuous variables and chi-square tests were used to compare those of categorical variables

*Los* length of stay

^&^Other mainly included Black, Hispanic, Asian, etc

*Significant difference between patients with delirium with history of fall without history of fall (p < 0.05)

### Associations between history of falls and the risks of delirium, urinary tract infection, and pressure injury

Table 2 shows an association between a history of falls and the risk of adverse outcomes in ICU patients. According to the results of the multivariate logistic regression analysis performed, compared to patients without a history of falls, those with such a history had a significantly increased prevalence of delirium during their critical illness, with an adjusted odds ratio (OR) of 2.11 (95% CI: 1.97–2.26). In addition, patients with a history of falls were also at risk for urinary tract infection and pressure injury, with adjusted odds ratios (ORs) of 1.50 (95% CI: 1.40–1.62) and 1.36 (95% CI: 1.26–1.47), respectively. These findings suggested that a history of falls could be an independent risk factor for delirium, UTIs, and pressure injury in the ICU.

**Table 2:** Logistic Regression: Association Between History of falls and Primary/Secondary Study Outcomes

	Without history of falls	History of falls	P-value	E-value (lower limit of the 95% CIs)
		ORs (95% CIs)		
Delirium^●^				
Unadjusted	Reference	2.27 (2.14, 2.41)	< 0.001*	Not applicable
Adjusted	Reference	2.11 (1.97, 2.26)	< 0.001*	2.26 (2.16)
Urinary tract infection^&^				
Unadjusted	Reference	1.75 (1.63, 1.88)	< 0.001*	Not applicable
Adjusted	Reference	1.50 (1.40, 1.62)	< 0.001*	1.75 (1.65)
Pressure injury^&^				
Unadjusted	Reference	1.52 (1.42, 1.64)	< 0.001*	Not applicable
Adjusted	Reference	1.36 (1.26, 1.47)	< 0.001*	1.61 (1.49)

*ORs* odds ratios, *CIs* confidence intervals

Logistic regression models were used to calculate odds ratios (ORs) with 95% confidence intervals (CIs)

^*^Significant difference between older adults with history of falls and without history of falls (p < 0.05)

^●^Delirium was adjusted for age, sex, race, marital status, dementia, depression, cerebrovascular disease, sedatives, CCI, SOFA, GCS and Braden score

^&^Aspiration pneumonia and pressure Injury were adjusted for age, sex, race, GCS, CCI, SOFA and Braden score

**Fig. 2:**
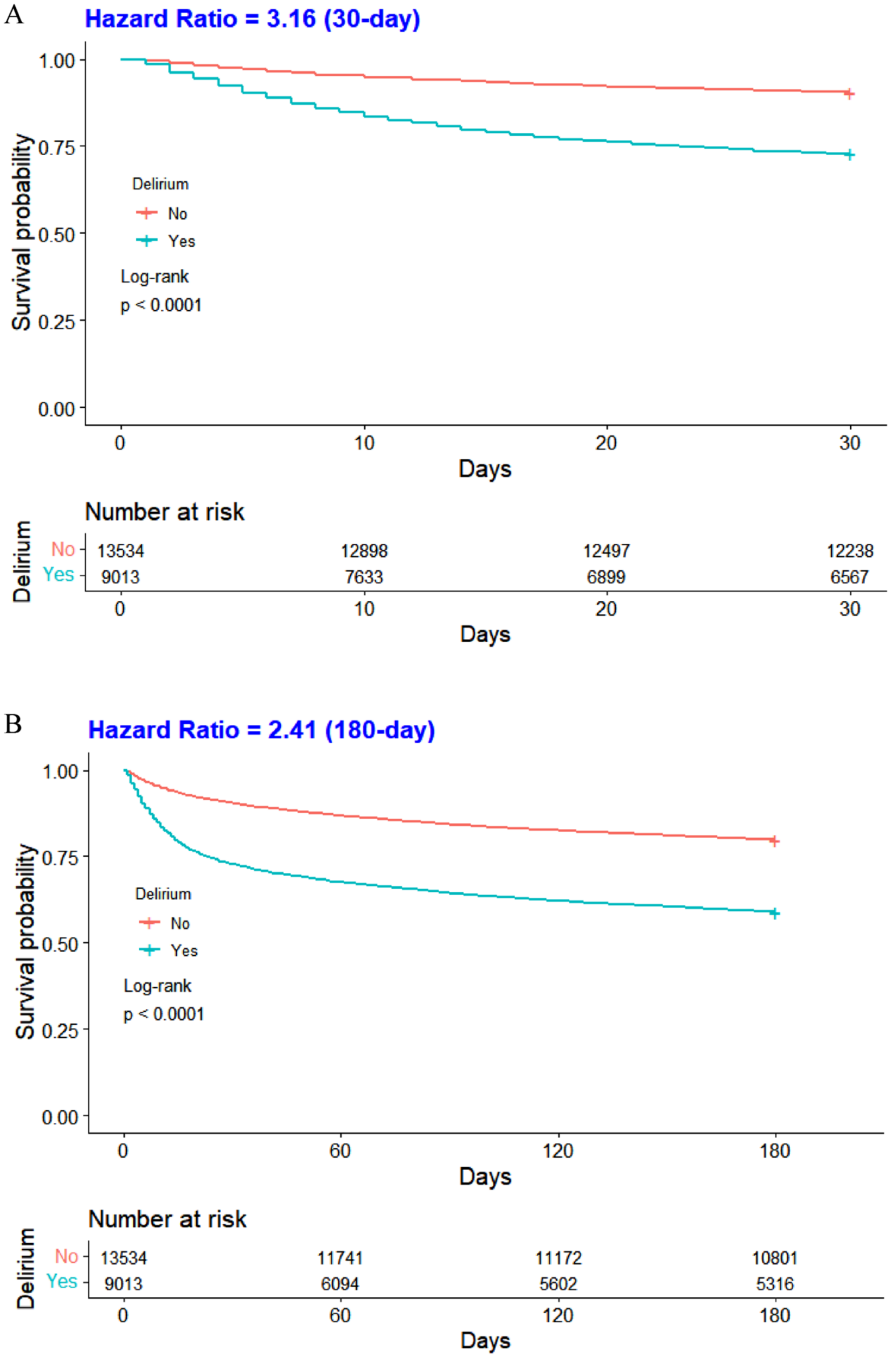

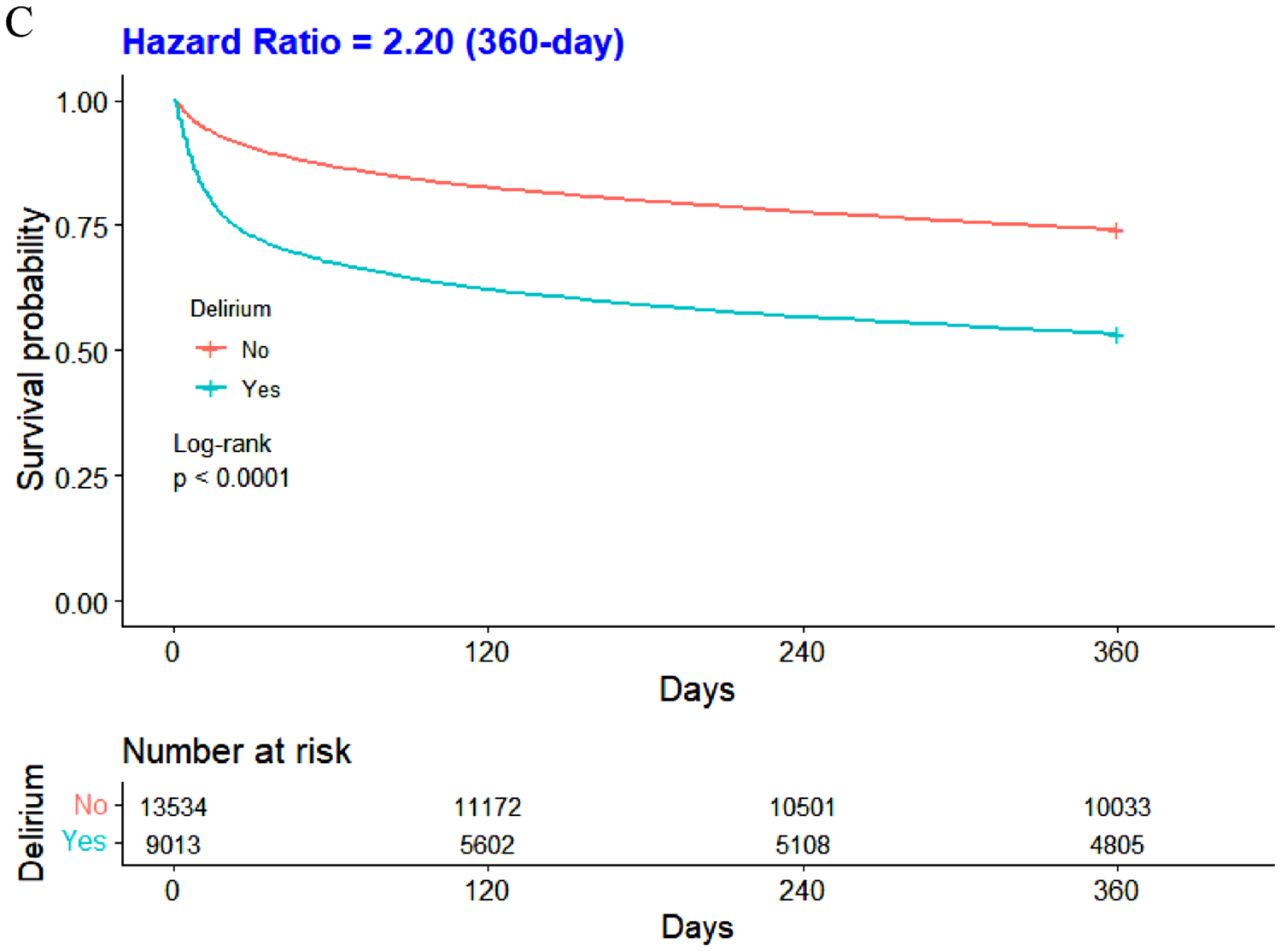
Comparison of Survival Rates of ICU Patients with and without Delirium at 30 (A), 180 (B), and 360 (C)-day Intervals

In addition, this study compared the survival rates of geriatric critically ill patients, both with and without delirium, at 30, 180, and 360-day intervals (Fig. 2A,2B,2C). The Kaplan–Meier survival curve demonstrated that elderly patients who developed delirium had significantly lower survival rates at 30, 180, and 360 days compared to those who without delirium (all p < 0.001).

### Subgroup analysis

**Fig. 3:**
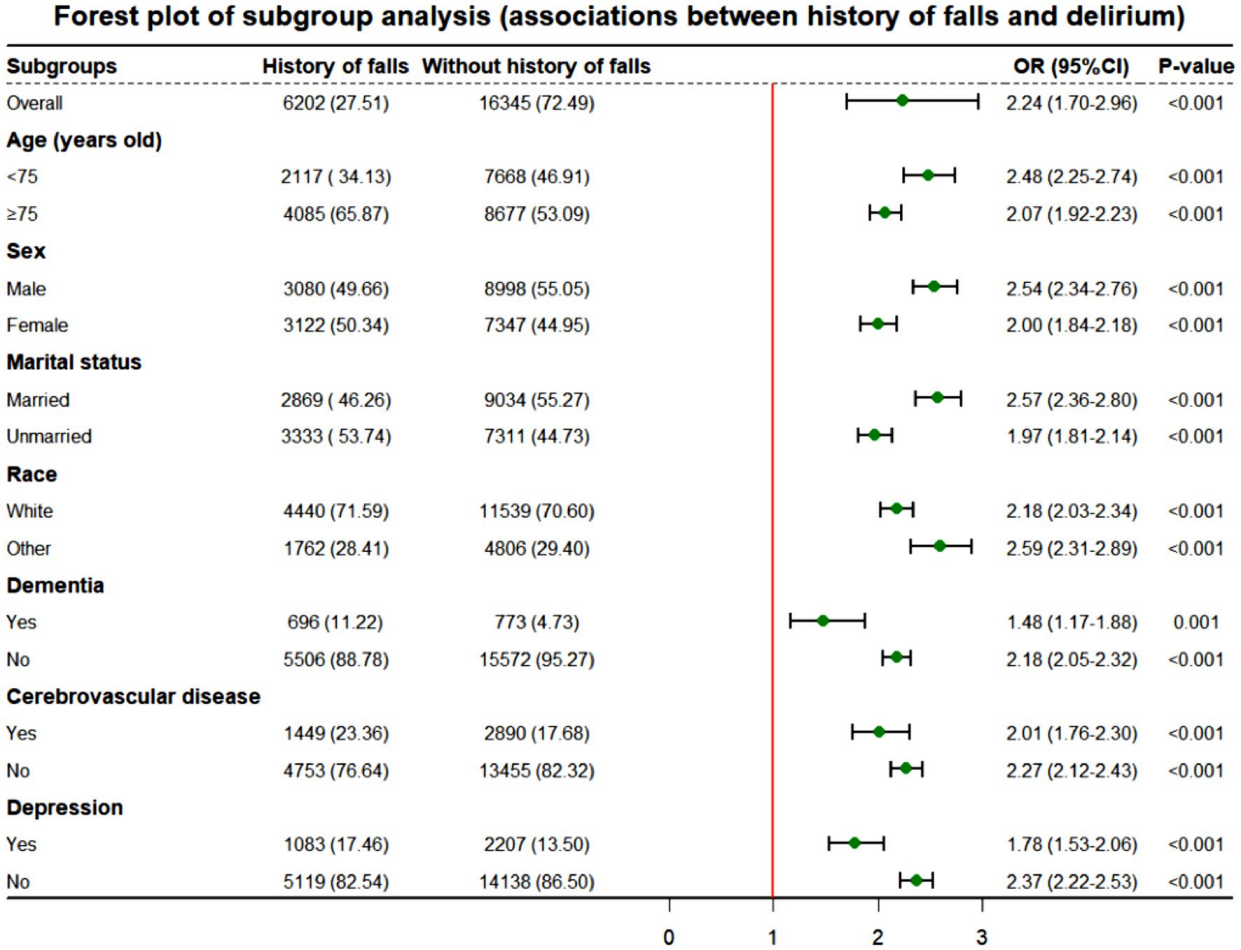
Subgroup analysis of fall history and delirium

In this study, a subgroup analysis elucidated the relationship between exposure and the primary outcome (delirium) to further our understanding of the association between a history of falls and delirium within subpopulations. The results show substantial statistical variation across all subgroups, suggesting a persistent association between a history of falls and the incidence of delirium within these boundaries (Fig. 3). Apparently, a history of falls may serve as a critical risk factor for delirium in ICU patients, with these findings applicable to individuals across different sex and age groups.

### Joint association

This study investigated the combined impact of a history of falls and delirium on mortality rates at 30, 180, and 360 days, presented in eTable 3 [Supplementary materials]. Baseline characteristics of the four patient cohorts were detailed in eTable 2 [Supplementary materials], highlighting differences between groups. The multivariate Cox proportional hazards model demonstrated that, compared to older patients without falls and delirium, those with both conditions had the highest mortality risk at 30, 180, and 360 days, with adjusted HRs of 4.07 (3.72, 4.45), 3.07 (2.87, 3.28), and 2.79 (2.62, 2.97), respectively.

### Sensitivity analysis

After PSM, most baseline parameters showed no significant differences between the two cohorts. Univariate and multivariate logistic regression analyses on the matched cohort yielded results consistent with the original population (eTable 2 in Supplementary Materials). This underscores the association between a history of falls and delirium, urinary tract infection, and pressure injury in elderly patients during critical illness (all p < 0.05). These outcomes emphasize the robustness and stability of our findings.

## Discussion

This study utilized publicly available MIMIC-IV data from the United States to investigate the association between falls within the previous 3 months and adverse outcomes in ICU patients, focusing on short-term mortality in elderly patients with delirium. Findings revealed that a history of falls within three months before hospital admission heightened the risk of delirium. These results align with prior research [23, 24]. A possible explanation may be that falls in older adults typically signal a decline in physical and cognitive function, as evidenced by impaired attention, executive function, and slowed gait [25]. A one-point decline in the Mini-Mental State Examination score corresponded to a subsequent 20% increase in the risk of falling [3]. Falls could also induce brain injury and physical trauma, leading to pain, extended hospital stays, and intensified treatment, all factors accelerating delirium onset [13, 26]. Identifying and addressing fall-related risk factors is crucial in preventing delirium in ICU patients. Healthcare professionals can utilize a patient's fall history before admission as an indicator for potential delirium, informing targeted interventions alongside other high-risk factors.

In the ICU setting, the interplay between a history of falls and the onset of UTIs and pressure injuries is multifaceted. Findings indicated an increased likelihood of pressure injuries in patients with falls, consistent with prior research [27]. The explanation lies in fall-related injuries restricting mobility, reducing turning frequency, and predisposing patients to prolonged pressure, thereby compromising skin integrity and fostering pressure ulcers [28]. Falls, causing brain damage and functional decline, often result in compromised bowel control, contributing to pressure injuries around the buttocks due to combined effects of urine, fecal stimulation, and pressure [29]. This research also discovered a correlation between a history of falls and urinary tract infections, aligning with the conclusions of prior studies [30]. Falls in older individuals elevate UTI risks through injuries necessitating catheterization, bacterial colonization, urethral mucosa damage, and a weakened immune response. Reduced mobility, often a consequence of falls, necessitates extended periods of immobilization and potentially the use of urinary catheters, both of which significantly elevate the risk of UTIs. Concurrently, immobilization increases the likelihood of developing pressure injuries due to sustained pressure on certain body areas. In ICUs, complex care protocols such as sedation and the use of physical restraints may inadvertently reduce patient mobility, thereby increasing the likelihood of UTIs and pressure injuries. It is crucial for ICU healthcare professionals to prioritize the prevention of these conditions through careful monitoring and the implementation of specific interventions. Strategies such as frequent repositioning and meticulous urinary catheter management are essential to minimize these risks and improve patient outcomes.

Delirium emerged as an independent risk factor for heightened mortality risk in this study. Patients developing delirium exhibited significantly reduced survival rates at 30, 180, and 360 days compared to their non-delirium counterparts, aligning with prior research [31]. Delirium represents an episode of acute brain failure and its damage to the brain may not be fully reversible and is closely associated with long-term cognitive decline [32], which can severely impact a patient's quality of life. Delirium onset is linked with inflammation and neuronal apoptosis, potentially leading to brain atrophy and diminished survival [33]. Concurrently, delirium-associated complications like aspiration pneumonia and increased restraint use extended hospital stays, elevating ICU infection risks and acting as an independent mortality risk factor [34]. Each additional hour of delay in hospitalization raised the risk of death by approximately 3% [35]. The joint effects unveiled a substantial mortality increase at 30, 180, and 360 days in patients with both a history of falls and delirium compared to those without. This survival analysis is pivotal for filling the research void by providing concrete data on mortality risks at critical junctures post-admission, thereby offering a clearer guide for clinical strategies aimed at reducing fall-related fatalities among ICU patients.

Our research demonstrates that a history of falls within three months prior to ICU admission significantly increases the likelihood of delirium, decreases survival rates, and elevates the incidence of secondary outcomes such as pressure ulcers and urinary tract infections in ICU patients. This highlights the critical need for early screening and intervention strategies focusing on fall history to mitigate these risks. By prioritizing fall history in patient evaluations, healthcare professionals can identify high-risk individuals for targeted preventive measures, thereby improving patient care and outcomes in the ICU.

This study boasts several strengths. Firstly, the substantial sample size enhances statistical precision, reducing variance and uncertainty in research findings. Secondly, the innovative assessment method, utilizing the CAM-ICU scale and nursing notes, ensures result stability and reliability. Thirdly, sensitivity analyses confirm the robustness of conclusions. Lastly, explicit definition of fall timing in the Morse Fall Scale score and identification of falls within three months quantify fall history, pinpointing associated high-risk factors [36]. These strengths significantly bolster the study's credibility.

There are also several limitations to the study. Firstly, its retrospective nature may lead to selection and information biases. Secondly, relying on nursing notes for assessing falls and delirium could result in underreporting. Lastly, the findings from a single tertiary academic medical center in Boston might not be broadly applicable to other settings. To overcome these limitations, future research should adopt a prospective study design could minimize biases associated with retrospective analyses. In addition, expanding the study to include multiple centers with diverse patient populations would improve the generalizability of the findings. Finally, integrating quantitative tools alongside nursing notes could offer a more comprehensive evaluation of falls and delirium, enriching the data quality.

## Conclusion

This study demonstrates that a history of falls significantly influences the onset of delirium in elderly ICU patients, also increasing urinary tract infections and pressure injuries. Moreover, patients with delirium exhibited significantly lower short-term survival rates than their healthy counterparts, posing a substantial threat to patient health. These findings highlight the need for healthcare professionals to adopt predictive measures and targeted interventions to enhance patient care and reduce costs, potentially guiding future research to refine management and preventive strategies for delirium and related conditions in the ICU.

### Supplementary Information

Below is the link to the electronic supplementary material.

Supplementary file1 (DOCX 47 KB)

## Declarations

### Data Availability Statement

Data are openly available in a public repository that can be accessed upon completion of the Protecting Human Research Participants web-based training from the National Institutes of Health. The datasets generated and analyzed during the current study are available from the corresponding authors, Jun Lyu and Qi-yuan Lyu, upon reasonable request.
