# Supplementary material for: Effect of a fall within three months of admission on delirium in critically Ill elderly patients: a population-based cohort study
Source: Aging Clin Exp Res. 2024 May 14;36(1):111. doi: 10.1007/s40520-024-02740-8 (PMC11093843; doi:10.1007/s40520-024-02740-8)
Supplement: Supplementary file 1 — Supplementary file1 (DOCX 47 KB) [file 40520_2024_2740_MOESM1_ESM.docx]

**Table 1B** **Univariate Analysis of Comorbidities Characteristics with and without History of Fall.**

| Variable | Overall (n=22547) | Without history of falls  (n=16345) | History of falls  (n=6202) | P-value |
| --- | --- | --- | --- | --- |
| **Comorbidities** |  |  |  |  |
| Myocardial infarct (%) |  |  |  | <0.001 |
| Yes/ No | 5129/17418 (22.7/77.3) | 3839/12506 (23.5/76.5) | 1290/4912 (20.8/79.2) |  |
| Congestive heart failure (%) |  |  |  | 0.464 |
| Yes/ No | 8767/13780 (38.9/61.1) | 6331/10014 (38.7/61.3) | 2436/3766 (39.3/60.7) |  |
| Dementia (%) |  |  |  | <0.001 |
| Yes/ No | 1469/21078 (6.5/93.5) | 773/15572 (4.7/95.3) | 696/5506 (11.2/88.8) |  |
| Chronic pulmonary disease (%) |  |  |  | 0.077 |
| Yes/ No | 6596/15951 (29.3/70.7) | 4836/11509 (29.6/70.4) | 1760/4442 (28.4/71.6) |  |
| Rheumatic disease (%) |  |  |  | 0.017 |
| Yes/ No | 995/21552 (4.4/95.6) | 688/15657 (4.2/95.8) | 307/5895 (5.0/95.0) |  |
| Mild liver disease (%) |  |  |  | 0.502 |
| Yes/ No | 1603/20944 (7.1/92.9) | 1150/15195 (7.0/93.0) | 453/5749 (7.3/92.7) |  |
| Diabetes (%) |  |  |  | 0.037 |
| Yes/ No | 7816/14731 (34.7/65.3) | 5733/10612 (35.1/64.9) | 2083/4119 (33.6/66.4) |  |
| Renal disease (%) |  |  |  | 0.011 |
| Yes/ No | 6421/16126 (28.5/71.5) | 4577/11768 (28.0/72.0) | 1844/4358 (29.7/70.3) |  |
| Malignant cancer (%) |  |  |  | <0.001 |
| Yes/ No | 3233/19314 (14.3/85.7) | 2464/13881 (15.1/84.9) | 769/5433 (12.4/87.6) |  |
| Severe liver disease (%) |  |  |  | 0.061 |
| Yes/ No | 618/21929 (2.7/97.3) | 427/15918 (2.6/97.4) | 191/6011 (3.1/96.9) |  |
| Cerebrovascular disease (%) |  |  |  | <0.001 |
| Yes/ No | 4339/18208 (19.2/80.8) | 2890/13455 (17.7/82.3) | 1449/4753 (23.4/76.6) |  |
| Hypertension (%) |  |  |  | 0.004 |
| Yes/ No | 10545/12002 (46.8/53.2) | 7741/8604 (47.4/52.6) | 2804/3398 (45.2/54.8) |  |
| Sepsis (%) |  |  |  | 0.001 |
| Yes/ No | 11889/10658 (52.7/47.3) | 8511/7834 (52.1/47.9) | 3378/2824 (54.5/45.5) |  |
| Depression (%) |  |  |  | <0.001 |
| Yes/ No | 3290/19257 (14.6/85.4) | 2207/14138 (13.5/86.5) | 1083/5119 (17.5/82.5) |  |
| Pain (%) |  |  |  | 0.009 |
| Yes/ No | 7151/15396 (31.7/68.3) | 5150/11195 (31.5/68.5) | 2001/4201 (32.3/67.7) |  |
| Paraplegia (%) |  |  |  | <0.001 |
| Yes/ No | 1429/21118 (6.3/93.7) | 843/15502 (5.2/94.8) | 586/5616 (9.4/90.6) |  |
| Peripheral vascular disease (%) |  |  |  | <0.001 |
| Yes/ No | 3472/19075 (15.4/84.6) | 2674/13671 (16.4/83.6) | 798/5404 (12.9/87.1) |  |
| Metastatic solid tumor (%) |  |  |  | 0.001 |
| Yes/ No | 1397/21150 (6.2/93.8) | 1068/15277 (6.5/93.5) | 329/5873 (5.3/94.7) |  |

**Table 1C Univariate Analysis of Treatment Characteristics with and without History of Fall.**

| Variable | Overall (n=22547) | Without history of falls  (n=16345) | History of falls  (n=6202) | P-value |
| --- | --- | --- | --- | --- |
| **Treatments and drugs** |  |  |  |  |
| Enteral nutrition (%) |  |  |  | <0.001 |
| Yes/ No | 371/1467 (20.2/79.8) | 287/1307 (18.0/82.0) | 84/160 (34.4/65.6) |  |
| Mechanical thrombectomy (%) |  |  |  | 0.233 |
| Yes/ No | 244/1594 (13.3/86.7) | 218/1376 (13.7/86.3) | 26/218 (10.7/89.3) |  |
| Dialysis present (%) |  |  |  | 0.001 |
| Yes/ No | 845/21702 (3.7/96.3) | 604/15741 (3.7/96.3) | 241/5961 (3.9/96.1) |  |
| Vaso (%) |  |  |  | <0.001 |
| Yes/ No | 8122/14425 (36.0/64.0) | 74/1520 (4.6/95.4) | 11/233 (4.5/95.5) |  |
| ^Sedatives (%) |  |  |  | <0.001 |
| Yes/ No | 10185/12362 (45.2/54.8) | 7910/8435 (48.4/51.6) | 2275/3927 (36.7/63.3) |  |
| IMV (%) |  |  |  | <0.001 |
| Yes/ No | 6352/16195 (28.2/71.8) | 4960/11385 (30.3/69.7) | 1392/4810 (22.4/77.6) |  |

**Table 1D Univariate Analysis of Clinical Parameters, Laboratory Parameters and Scoring Features with and without History of Fall.**

| Variable | Overall (n=22547) | Without history of falls  (n=16345) | History of falls  (n=6202) | P-value |
| --- | --- | --- | --- | --- |
| **Scores** | | | | |
| GCS | 14.00 (11.00, 15.00) | 14.00 (12.00, 15.00) | 13.00 (10.00, 14.00) | <0.001 |
| SOFA | 5.00 (3.00, 7.00) | 5.00 (3.00, 7.00) | 4.00 (3.00, 7.00) | 0.568 |
| Braden score | 15.00 (13.00, 17.00) | 15.00 (13.00, 17.00) | 15.00 (13.00, 16.00) | <0.001 |
| CCI | 7.00 (5.00, 9.00) | 7.00 (5.00, 9.00) | 7.00 (5.00, 9.00) | <0.001 |
| **Clinical Parameters** |  |  |  |  |
| Heart rate mean | 80.96 (71.96, 91.57) | 80.97 (72.33, 91.38) | 80.90 (70.91, 92.00) | 0.078 |
| MBP mean (mmHg) | 75.41 (69.64, 82.76) | 75.04 (69.55, 81.97) | 76.52 (69.93, 84.84) | <0.001 |
| Respiratory rate mean | 18.77 (16.77, 21.29) | 18.71 (16.71, 21.22) | 18.90 (16.94, 21.42) | <0.001 |
| Temperature mean (℃) | 36.78 (36.59, 37.00) | 36.77 (36.59, 36.99) | 36.81 (36.62, 37.04) | <0.001 |
| **Laboratory parameters** |  |  |  |  |
| SpO_2_ mean (%) | 97.00 (95.63, 98.28) | 97.03 (95.64, 98.29) | 96.93 (95.60, 98.26) | 0.122 |
| Albumin (g/dL) | 3.50 (3.00, 4.00) | 3.50 (3.00, 4.00) | 3.40 (2.90, 3.90) | <0.001 |
| BUN (mg/dL) | 22.00 (15.00, 36.00) | 21.00 (15.00, 34.00) | 24.00 (17.00, 39.00) | <0.001 |
| Creatinine (mg/dL) | 1.00 (0.80, 1.50) | 1.00 (0.80, 1.50) | 1.10 (0.80, 1.60) | <0.001 |
| Bun crea (mg/dL) | 20.67 (16.00, 27.00) | 20.00 (15.88, 26.36) | 21.43 (16.67, 28.33) | <0.001 |
| Neutrophils | 79.50 (70.70, 86.10) | 79.10 (70.30, 85.90) | 80.50 (71.60, 86.70) | <0.001 |
| lymphocytes | 11.60 (6.60, 18.80) | 12.00 (6.90, 19.30) | 10.70 (6.10, 17.30) | <0.001 |
| NLR | 6.77 (3.74, 12.72) | 6.53 (3.61, 12.40) | 7.40 (4.14, 13.75) | <0.001 |

Abbreviations: GCS: Glasgow Coma Score; SOFA: sequential organ failure assessment; IMV: invasive mechanical ventilation; Vaso: vasoactive substance; CCI: Charlson comorbidity index

Note:

^Sedatives mainly included benzodiazepines, propofol and dexmedetomidine;

*Significant difference between patients with delirium with history of fall without history of fall (p < 0.05).

Median and interquartile range (25th and 75th percentiles) were computed for continuous variables, and frequencies and percentages were computed for categorical variables.

The Wilcoxon rank-sum test was used to compare group differences for continuous variables and chi-square tests were used to compare those of categorical variables.

**e Table 2 Analysis of the joint effect of fall history and delirium**

| Variable | Without fall and delirium  (n=10708) | Fall without delirium  (n=2826) | Delirium without fall  (n=5637) | Fall and delirium  (n=3376) | P-value |
| --- | --- | --- | --- | --- | --- |
| **General characteristics** |  |  |  |  |  |
| Age (years old) | 75.20 [69.87, 81.85] | 79.28 [72.04, 86.49] | 76.88 [70.99, 83.82] | 79.81 [72.43, 86.82] | <0.001 |
| Gender (%) |  |  |  |  | <0.001 |
| Male | 6031 (56.3) | 1369 (48.4)) | 2967 (52.6) | 1711 (50.7) |  |
| Female | 4677 (43.7) | 1457 (51.6) | 2670 (47.4) | 1665 (49.3) |  |
| Marital status |  |  |  |  | <0.001 |
| Married | 6119 (57.1) | 1289 (45.6) | 2915 (51.7) | 1580 (46.8) |  |
| Unmarried/unknown | 4589 (42.9) | 1537 (54.4) | 2722 (48.3) | 1796 (53.2) |  |
| Race (%) |  |  |  |  | <0.001 |
| White | 7763 (72.5) | 2157 (76.3) | 3776 (67.0) | 2283 (67.6) |  |
| Others^&^ | 2945 (27.5) | 669 (23.7) | 1861 (33.0) | 1093 (32.4) |  |
| Language (%) |  |  |  |  | 0.266 |
| English | 9539 (89.1) | 2535 (89.7) | 4980 (88.3) | 2999 (88.8) |  |
| Unknown | 1169 (10.9) | 291 (10.3) | 657 (11.7) | 377 (11.2) |  |
| Weight (kg) | 78.50 [66.50, 91.85] | 73.85 [62.00, 86.70] | 76.30 [64.00, 90.60] | 72.00 [60.14, 85.70] | <0.001 |
| Hospital Los (days) | 6.75 [4.55, 10.15] | 6.84 [4.47, 10.60] | 9.83 [6.02, 16.59] | 8.90 [5.31, 15.07] | <0.001 |
| ICU Los (days) | 2.04 [1.36, 3.13] | 2.05 [1.47, 3.20] | 3.88 [2.17, 7.32] | 3.35 [1.97, 6.13] | <0.001 |
| **Scores** |  |  |  |  |  |
| GCS | 15.00 [14.00, 15.00] | 14.00 [14.00, 15.00] | 12.00 [8.00, 14.00] | 12.00 [8.00, 13.25] | <0.001 |
| SOFA | 4.00 [2.00, 6.00] | 4.00 [2.00, 6.00] | 6.00 [4.00, 9.00] | 5.00 [3.00, 8.00] | <0.001 |
| Braden score | 15.00 [14.00, 17.00] | 15.00 [14.00, 17.00] | 14.00 [12.00, 16.00] | 14.00 [12.00, 16.00] | <0.001 |
| CCI | 6.00 [5.00, 8.00] | 7.00 [5.00, 9.00] | 7.00 [6.00, 9.00] | 7.00 [5.00, 9.00] | <0.001 |
| **Comorbidities** |  |  |  |  |  |
| Myocardial infarct (%) |  |  |  |  | <0.001 |
| Yes | 2568 (24.0) | 627 (22.2) | 1271 (22.5) | 663 (19.6) |  |
| No | 8140 (76.0) | 2199 (77.8) | 4366 (77.5) | 2713 (80.4) |  |
| Congestive heart failure (%) |  |  |  |  | 0.464 |
| Yes | 4056 (37.9) | 1179 (41.7) | 2275 (40.4) | 1257 (37.2) |  |
| No | 6652 (62.1) | 1647 (58.3) | 3362 (59.6) | 2119 (62.8) |  |
| Dementia (%) |  |  |  |  | <0.001 |
| Yes | 228 (2.1) | 153 (5.4) | 545 (9.7) | 543 (16.1) |  |
| No | 10180 (97.9) | 2673 (94.6) | 5092 (90.3) | 2833 (83.9) |  |
| Chronic pulmonary disease (%) |  |  |  |  | <0.001 |
| Yes | 3122 (29.2) | 890 (31.5) | 1714 (30.4) | 870 (25.8) |  |
| No | 7586 (70.8) | 1936 (68.5) | 3923 (69.6) | 2506 (74.2) |  |
| Rheumatic disease (%) |  |  |  |  | 0.029 |
| Yes | 472 (4.4) | 144 (5.1) | 216 (3.8) | 163 (4.8) |  |
| No | 10236 (95.6) | 2682 (94.9) | 5421 (96.2) | 3213 (95.2) |  |
| Mild liver disease (%) |  |  |  |  | <0.001 |
| Yes | 661 (6.2) | 195 (6.9) | 189 (8.7) | 258 (7.6) |  |
| No | 10047 (93.8) | 2631 (93.1) | 5148 (91.3) | 3118 (92.4) |  |
| Diabetes (%) |  |  |  |  | <0.001 |
| Yes | 3585 (33.5) | 964 (34.1) | 2148 (38.1) | 1119 (33.1) |  |
| No | 7123 (66.5) | 1862 (65.9) | 3489 (61.9) | 2257 (66.9) |  |
| Renal disease (%) |  |  |  |  | <0.001 |
| Yes | 2859 (26.7) | 836 (29.6) | 1718 (30.5) | 1008 (29.9) |  |
| No | 7849 (73.3) | 1990 (70.4) | 3919 (69.5) | 2368 (70.1) |  |
| Malignant cancer (%) |  |  |  |  | <0.001 |
| Yes | 1642 (15.3) | 391 (13.8) | 822 (14.6) | 378 (11.2) |  |
| No | 9066 (84.7) | 2435 (86.2) | 4815 (85.4) | 2998 (88.8) |  |
| Severe liver disease (%) |  |  |  |  | <0.001 |
| Yes | 200 (1.9) | 85 (3.0) | 227 (4.0) | 106 (3.1) |  |
| No | 10508 (98.1) | 2741 (97.0) | 5410 (96.0) | 3270 (96.9) |  |
| Cerebrovascular disease (%) |  |  |  |  | <0.001 |
| Yes | 1394 (13.0) | 459 (16.2) | 1496 (26.5) | 990 (29.3) |  |
| No | 9314 (87.0) | 2367 (83.8) | 4141 (73.5) | 2386 (70.7) |  |
| Hypertension (%) |  |  |  |  | <0.001 |
| Yes | 5191 (48.5) | 1272 (45.0) | 2550 (45.2) | 1532 (45.4) |  |
| No | 5517 (51.5) | 1554 (55.0) | 3087 (54.8) | 1844 (54.6) |  |
| Sepsis (%) |  |  |  |  | <0.001 |
| Yes | 4644 (43.4) | 1255 (44.4) | 3867 (68.6) | 2123 (62.9) |  |
| No | 6064 (56.6) | 1571 (55.6) | 1770 (31.4) | 1253 (37.1) |  |
| Depression (%) |  |  |  |  | <0.001 |
| Yes | 1344 (12.6) | 506 (17.9) | 863 (15.3) | 577 (17.1) |  |
| No | 9364 (87.4) | 2320 (82.1) | 4774 (84.7) | 2799 (82.9) |  |
| Pain (%) |  |  |  |  | <0.001 |
| Yes | 3596 (33.6) | 1062 (37.6) | 1554 (27.6) | 939 (27.8) |  |
| No | 7112 (66.4) | 1764 (62.4) | 4083 (72.4) | 2437 (72.2) |  |
| Paraplegia (%) |  |  |  |  | <0.001 |
| Yes | 335 (3.1) | 152 (5.4) | 508 (9.0) | 434 (12.9) |  |
| No | 10373 (96.9) | 2674 (94.6) | 5129 (91.0) | 2942 (87.1) |  |
| Peripheral vascular disease (%) |  |  |  |  | <0.001 |
| Yes | 1707 (15.9) | 391 (13.8) | 967 (17.2) | 407 (12.1) |  |
| No | 9001 (84.1) | 2435 (86.2) | 4670 (82.8) | 2969 (87.9) |  |
| Metastatic solid tumor (%) |  |  |  |  | <0.001 |
| Yes | 730 (6.8) | 168 (5.9) | 338 (6.0) | 161 (4.8) |  |
| No | 9978 (93.2) | 2658 (94.1) | 5299 (94.0) | 3215 (95.2) |  |
| **Clinical Parameters** |  |  |  |  |  |
| Heart rate mean | 80.19 [71.91, 89.70] | 79.75 [70.44, 90.75] | 82.85 [73.18, 94.65] | 81.68 [71.48, 92.98] | <0.001 |
| MBP mean (mmHg) | 74.86 [69.48, 81.57] | 75.17 [68.76, 83.30] | 75.32 [69.68, 82.65] | 77.77 [70.90, 86.10] | <0.001 |
| Respiratory rate mean | 18.54 [16.60, 20.88] | 18.74 [16.81, 21.26] | 19.08 [16.95, 21.75] | 19.04 [17.04, 21.60] | <0.001 |
| Temperature mean (℃) | 36.75 [36.58, 36.93] | 36.75 [36.58, 36.94] | 36.83 [36.61, 37.12] | 36.86 [36.65, 37.14] | <0.001 |
| **Laboratory parameters** |  |  |  |  |  |
| SpO_2_ mean (%) | 96.91 [95.55, 98.13] | 96.67 [95.38, 97.88] | 97.27 [95.81, 98.55] | 97.16 [95.79, 98.52] | <0.001 |
| Albumin (g/dL) | 3.60 [3.10, 4.10] | 3.50 [2.90, 3.90] | 3.40 [2.80, 3.90] | 3.40 [2.90, 3.80] | <0.001 |
| BUN (mg/dL) | 20.00 [15.00, 32.00] | 24.00 [16.00, 39.00] | 24.00 [16.00, 39.00] | 24.00 [17.00, 39.00] | <0.001 |
| Creatinine (mg/dL) | 1.00 [0.80, 1.40] | 1.10 [0.80, 1.60] | 1.10 [0.80, 1.70] | 1.10 [0.80, 1.60] | <0.001 |
| Bun crea (mg/dL) | 20.00 [16.00, 25.85] | 21.43 [16.67, 28.43] | 20.91 [15.83, 27.50] | 21.33 [16.36, 28.10] | <0.001 |
| Neutrophils | 78.30 [69.50, 85.00] | 80.00 [71.10, 86.40] | 80.80 [72.20, 87.00] | 81.00 [72.00, 87.00] | <0.001 |
| lymphocytes | 13.10 [7.60, 20.40] | 11.20 [6.50, 18.00] | 10.00 [5.80, 16.90] | 10.20 [5.90, 16.70] | <0.001 |
| NLR | 5.92 [3.41, 11.08] | 7.00 [3.94, 13.01] | 7.93 [4.31, 14.71] | 7.80 [4.31, 14.50] | <0.001 |
| **Treatments and drugs** |  |  |  |  |  |
| Dialysis present (%) |  |  |  |  | <0.001 |
| Yes | 305 (2.8) | 110 (3.9) | 299 (5.3) | 131 (3.9) |  |
| No | 10403 (97.2) | 2716 (96.1) | 5338 (94.7) | 3245 (96.1) |  |
| Vaso (%) |  |  |  |  | <0.001 |
| Yes | 3926 (36.7) | 674 (23.8) | 2499 (44.3) | 1023 (30.3) |  |
| No | 6782 (63.3) | 2152 (76.2) | 3138 (55.7) | 2353 (69.7) |  |
| Sedatives (%) |  |  |  |  | <0.001 |
| Yes | 4788 (44.7) | 748 (26.5) | 3122 (55.4) | 1527 (45.2) |  |
| No | 5920 (55.3) | 2078 (73.5) | 2515 (44.6) | 1849 (54.8) |  |
| IMV (%) |  |  |  |  | <0.001 |
| Yes | 2532 (23.6) | 347 (12.3) | 2426 (43.1) | 1045 (31.0) |  |
| No | 8176 (76.4) | 2479 (87.7) | 3209 (56.9) | 2331 (69.0) |  |
| **Outcomes** |  |  |  |  |  |
| Pressure injury (%) |  |  |  |  | <0.001 |
| Yes | 1176 (11.0) | 529 (18.7) | 1551 (27.5) | 920 (27.3) |  |
| No | 9532 (89.0) | 2297 (81.3) | 4086 (72.5) | 2456 (72.7) |  |
| Urinary tract infection (%) |  |  |  |  | <0.001 |
| Yes | 1327 (12.4) | 593 (21.0) | 1242 (22.0) | 931 (27.6) |  |
| No | 9381 (87.6) | 2233 (79.0) | 4395 (78.0) | 2445 (72.4) |  |
| 30-day mortality (%) |  |  |  |  | <0.001 |
| Alive | 9793 (91.5) | 2445 (86.5) | 4214 (74.8) | 2353 (69.7) |  |
| Expired | 915 (8.5) | 381 (13.5) | 1423 (25.2) | 1023 (30.3) |  |
| 180-day mortality (%) |  |  |  |  | \| <0.001 \| \| --- \| |
| Alive | 8760 (81.8) | 2041 (72.2) | 3453 (61.3) | 1863 (55.2) |  |
| Expired | 1948 (18.2) | 785 (27.8) | 2184 (38.7) | 1513 (44.8) |  |
| 360-day mortality (%) |  |  |  |  | <0.001 |
| Alive | 8203 (76.6) | 1830 (64.8) | 3141 (55.7) | 1664 (49.3) |  |
| Expired | 2505 (23.4) | 996 (35.2) | 2496 (44.3) | 1712 (50.7) |  |

Abbreviations: Los: length of stay; GCS: Glasgow Coma Score; SOFA: sequential organ failure assessment; IMV: invasive mechanical ventilation; Vaso: vasoactive substance; CCI: Charlson comorbidity index

Note:

Group 1: Neither of them (history of falls and delirium);

Group 2: History of falls alone;

Group 3: Delirium alone;

Group 4: Both of them (history of falls and delirium).

^&^Other mainly included Black, Hispanic, Asian, etc;

Median and interquartile range (25th and 75th percentiles) were computed for continuous variables, and frequencies and percentages were computed for categorical variables.

The Wilcoxon rank-sum test was used to compare group differences for continuous variables and chi-square tests were used to compare those of categorical variables.

**eTable 3** Joint associations of history of falls and delirium with 30-day, 180-day and 360-day mortality in older adults.

|  | Group 1 | Group 2 | Group 3 | Group 4 |
| --- | --- | --- | --- | --- |
|  |  | HRs (95% CIs) | | |
| 30-day mortality#  Model 1  Model 2 | Reference  Reference | 1.61 (1.43, 1.82)  1.43 (1.27, 1.61) | 3.27 (3.01, 3.55)  1.67 (1.53, 1.83) | 4.07 (3.72, 4.45)  2.15 (1.95, 2.36) |
| 180-day mortality#  Model 1  Model 2 | Reference  Reference | 1.61 (1.48, 1.75)  1.43 (1.32, 1.56) | 2.51 (2.36, 2.66)  1.48 (1.39, 1.58) | 3.07 (2.87, 3.28)  1.84 (1.71, 1.97) |
| 360-day mortality#  Model 1  Model 2 | Reference  Reference | 1.61 (1.50, 1.74)  1.45 (1.34, 1.56) | 2.28 (2.16, 2.41)  1.42 (1.34, 1.51) | 2.79 (2.62, 2.97)  1.76 (1.65, 1.87) |

Abbreviations: HRs: hazard ratios; CIs: confidence intervals.

Note:

Group 1: Neither of them (history of falls and delirium);

Group 2: History of falls alone;

Group 3: Delirium alone;

Group 4: Both of them (history of falls and delirium).

#Cox proportional hazards regression models were used to calculate hazard ratios (HRs) with 95% confidence intervals (CIs).

Model 1 was unadjusted;

Model 2 was adjusted for age, sex, race, GCS, SOFA score, Braden score and CCI.
